# DDIAS promotes endometrial cancer progression via β-catenin signaling

**DOI:** 10.1371/journal.pone.0331851

**Published:** 2025-10-06

**Authors:** Xiaojuan Tian, Menglu Liu, Jiangbo Zhao, Chusi Lao, Boya Liu, Yuping Suo

**Affiliations:** 1 The Fifth Clinical Medical College of Shanxi Medical University, Taiyuan, Shanxi, China; 2 Shanxi University Of Chinese Medicine, Taiyuan, Shanxi, China; Federal University of Rio de Janeiro: Universidade Federal do Rio de Janeiro, BRAZIL

## Abstract

**Background:**

DDIAS has been recognized as an oncogene in various cancers, but its role in endometrial cancer remains unexplored.

**Methods:**

The expression of DDIAS in normal endometrium and endometrial cancer samples was analysed via the The Cancer Genome Atlas (TCGA) database, and prognostic analysis was performed. The differential expression of DDIAS between endometrial cancer and normal endometrium tissues was analyzed using quantitative polymerase chain reaction (qPCR). To study the expression of DDIAS in endometrial cancer, immunohistochemistry was performed on endometrial cancer, atypical endometrial hyperplasia and normal endometrial tissue. The association between DDIAS expression and clinicopathology was analysed. The expression of DDIAS in endometrial cancer cell lines was studied via Western blot (WB) analysis. DDIAS was knocked down in endometrial cancer cell lines via small interfering RNA (siRNA), and the effects of DDIAS knockdown on endometrial cancer cell biology and its related regulatory mechanisms were investigated via Cell Counting Kit-8(CCK8), Colony formation assay, scratch test, Transwell, and WB assays. Finally, the relevant regulatory mechanisms were verified using **rescue** experiments.

**Results:**

According to the public database analysis, High DDIAS expression correlates with endometrial cancer and predicts unfavorable prognosis..The qPCR confirmed higher expression of *DDIAS* in tumor samples.We found that DDIAS was highly expressed in endometrial cancer and atypical endometrial hyperplasia, and that the upregulation of DDIAS expression predicted poor prognosis. In endometrial cancer, higher DDIAS expression was associated with increased tumor grade and advanced FIGO stage. In terms of cellular function, knocking down DDIAS suppressed the proliferation, migration and invasion capabilities of endometrial cancer cells. In the mechanistic pathway, reducing DDIAS expression led to the inhibition of β-catenin and its downstream targets, including c-Myc, cyclin D1, and survivin, while also suppressing epithelial-mesenchymal transition (EMT).However, these changes were rescued by the upregulation of β-catenin.

**Conclusion:**

DDIAS regulated EMT in endometrial cancer cell migration and invasion through the β-catenin pathway, demonstrating that DDIAS is a potential target for the treatment of endometrial cancer.

## 1. Introduction

Endometrial cancer (EC) ranks among the most common malignant tumors of the female reproductive tract globally, with the number of EC patients projected to rise to 122,000 by 2030 [1]. Patients with early-stage EC have a 5-year overall survival rate of over 74%, whereas the prognosis for advanced-stage EC is significantly worse. Specifically, the 5-year survival rate drops to around 48% for stage III patients and further declines to just 15% for those with stage IV disease [2]. As the aetiology of EC remains to be fully elucidated and effective treatment options for advanced and recurrent EC are limited, exploring new therapeutic targets and developing new screening methods is imperative.

DNA damage-induced apoptosis suppressor (DDIAS) is also known by the names nitric oxide inducible gene proteins (noxins) and chromosome 11 open reading frames (C11orf82) [3]. DDIAS is an antiapoptotic protein that acts as an agent in DNA repair reactions [4]. DDIAS exhibits high levels of expression in numerous cancers, including liver, lung, breast, and colorectal cancers, as well as gliomas [5–8]. The current study revealed that DDIAS is involved in the process of tumorigenesis and development, promotes the proliferation, invasion and migration of tumor cells in in vitro experiments, and is involved in tumor metastasis and drug resistance [9]. Recently, the relationship between DDIAS and the development of malignant tumours has received widespread attention, and DDIAS may serve as a novel therapeutic target. However, the role of DDIAS in EC is unknown.

The objective of the study was to analyse the expression and mechanism of action of DDIAS in EC. The expression of DDIAS in EC was first analysed via public databases, and a prognostic analysis was subsequently performed. Immunohistochemistry was performed to analyse DDIAS’s association with clinicopathological features and to assess its expression in tissue samples from 116 patients with endometrial cancer, 28 patients with atypical endometrial hyperplasia, and 28 patients with a normal endometrium. The protein expression levels of DDIAS in four common endometrial cancer cell lines were then analysed via WB. Finally, WB, CCK8, Colony formation assay, scratch, and Matrigel invasion assays were evaluated to determine the effects on cell proliferation, migration, invasion, and related pathways after DDIAS knockdown.

## 2. Materials & methods

### 2.1 Bioinformatic analysis

Gene expression profiles of 543 tumour samples and 35 normal samples with Overall Survival (OS) data were retrieved from the TCGA-UCEC database to analyse the expression of DDIAS mRNA in tumour and normal tissues. The R package (v. 4.1.0) “survminer” was used to group the high and low levels of DDIAS gene expression and to perform prognostic analysis. DDIAS protein expression differences in endometrial cancer and normal tissues were investigated in the Human Protein Atlas (HPA) database(https://www.proteinatlas.org/).

### 2.2 Patients and specimens

To explore the differential expression of DDIAS in clinical samples, we chose 10 patients with ECwho attended Shanxi Provincial People’s Hospital for surgical treatment from August 1, 2024 to August 31, 2024 and 10 patients who underwent surgery for other gynecological diseases during the same period, and collected endometrial cancer tissue samples and normal endometrial tissue samples, respectively. The samples were collected quickly after tissue exfoliation and immediately placed in liquid nitrogen tanks for preservation. Human tissue arrays were purchased from Shanghai Xinchao Company and Qingdao Spite Company (36 samples of endometrial cancer and 11 samples of corresponding paracancerous tissues, 80 samples of endometrial cancer and 31 samples of corresponding paracancerous tissues, respectively). In September 2024, we selected 28 patients with atypical endometrial hyperplasia who underwent surgical treatment and were diagnosed pathologically at Shanxi Provincial People’s Hospital from January 2021 to July 2024, and collected samples of their atypical hyperplastic endometrial hyperplasia. In September 2024, 28 normal endometrial samples were extracted from patients who underwent surgical excision for other gynecological treatments from September 2018 to March 2022 at Shanxi Provincial People’s Hospital. All 116 endometrial cancer samples were analysed for their histological subtype, degree of differentiation and tumour stage according to the 2009 International Federation of Gynaecology and Obstetrics (FIGO) [10]. The median age of the 172 patients was 45 years, ranging from 29 to 80 years. Among the 116 patients with endometrial cancer, 23 patients were <50 years old, and 92 patients were ≥50 years old. There were 77 stage I patients, 2 stage II patients, 8 stage III patients, and 2 stage IV patients. There were 18 cases of G1, 65 cases of G2 and 14 cases of G3. The size of the tumour was < 5 cm in 21 patients and ≥5 cm in 10 patients. The samples included 104 cases of adenocarcinoma, 2 cases of adenocarcinoma with mucinous differentiation, 8 cases of adenocarcinoma with squamous differentiation, 1 case of clear cell carcinoma, and 1 case of high-grade plasma carcinoma. Twenty-eight patients had atypical endometrial hyperplasia, 20 patients were <50 years old, and 8 patients were ≥50 years old. Basic information has been added in more detail (Table 1). This study was conducted with approval from the Ethics Committee of Shanxi Provincial People’s Hospital(Date: 07/22/2024/No: (2024)557), and informed consent was secured from all participating patients. All clinical studies were performed following the principles articulated in the Declaration of Helsinki.

**Table 1:** Patient clinical data.

Patient clinical data		Number of examples	proportion
Age	<50	23	20.00%
	≥50	92	80.00%
Histological type	Adenocarcinoma	104	89.66%
	Adenocarcinoma with mucinous differentiation	2	1.72%
	Adenocarcinoma with squamous differentiation	8	6.90%
	Clear cell carcinoma	1	0.86%
	High-grade serous carcinoma	1	0.86%
FIGO Staging	PhaseⅠ	77	86.52%
	PhaseⅡ	2	2.25%
	PhaseⅢ	8	8.99%
	PhaseⅣ	2	2.25%
Histological Grading	High differentiation (G1)	18	18.56%
	Medium differentiation (G2)	65	67.01%
	Low differentiation (G3)	14	14.43%
Tumor size	<5 cm	21	67.74%
	≥5 cm	10	32.26%

### 2.3 Quantitative polymerase chain reaction

Total RNA was extracted from all samples using TRIzol reagent (Ambion, Shanghai, China), and cDNA was synthesized with First-Strand cDNA synthesis kit (Servicebio, Wuhan, China). Prognostic gene primers, synthesized by Beijing Tsingke Biotech Co., Ltd. (Beijing, China), are detailed in **Online Resource 1**. qPCR was performed using the CFX96TM Real-Time PCR Detection System (BIO-RAD, U.S.A.). The 2-ΔΔCT method [11] with GAPDH as positive control was used to calculate relative gene expression levels.

### 2.4 Immunohistochemistry

Paraffin-embedded tissue samples were sliced into sections with a thickness of 4μm. Tissue paraffin sections were dewaxed with xylene, dehydrated with different concentrations of ethanol, and finally antigenically repaired with citrate antigen repair buffer. Tissue sections were treated with a murine monoclonal anti-DDIAS antibody (1:100; Abmart; Shanghai, China) in a wet box at 4°C overnight, and subsequently incubated with a goat anti-mouse IgG secondary antibody at room temperature for 30 minutes.After the tissue sections were washed with PBS buffer, they were colour developed with a DAB horseradish peroxidase colour development kit (Myxin Bio). Finally, the samples were restained with haematoxylin, subjected to dehydration using anhydrous ethanol, and sealed using neutral gum. Images were taken and the results were analysed via ImageJ software.

### 2.5 Cell lines and cell culture

The endometrial cancer cell KLE and HEC-1-A were obtained from Pricella (Wuhan, China), and HEC-1-B and Ishikawa were obtained from abmGood (Richmond, Canada). Human endometrial epithelial immortalized cells were obtained from Immocell (Xiamen, China). KLE cells were cultured in complete DMEM/F12 medium (HyClone, USA), HEC-1-A cells were cultured in complete Mccoy’s 5A medium (HyClone, USA), HEC-1-B cells and Ishikawa cells were cultured in complete RPMI-1640 medium (HyClone, USA), which contained 10% fetal bovine serum (Cellmax, Australia) and 1% streptomycin-penicillin (Beijing Sorabo Technology Co., Ltd., China). Human endometrial epithelial immortalized cells were cultured in a specialized medium (Immocell, China). All cells were cultured in a 37°C incubator with 5% CO_2_.

### 2.6 Small interfering RNA (siRNA) transfection

The siRNA-DDIAS and siRNA-NC used in this study were purchased from GenePharma, and the target sequences were as follows: siDDIAS#1, 5′-GGAACCAAUAAAGGUUUUAATT-3′; siDDIAS#2, 5′-CCUGCAUACACCACCUUAUATT-3′; siDDIAS#3, 5′-AGGUUAUUGCAGGCUUUACUTT-3′; siDDIAS#4, 5′-GACCAUUCUAGUCUAAAUATT-3′; siNC, 5′-UUCUCCGAACGUGUCACGUTT-3′. Cells were transfected with 500 nM siRNA using CALNPTM RNAi reagent (Dona) following the manufacturer’s protocol.

### 2.7 Cell Counting Kit-8(CCK8)

Ishikawa and KLE cells were seeded in 96-well plates at a density of 1 × 10³ cells/well. At 0, 24, 48, 72, and 96 hours post-seeding, 10 μL of CCK-8 solution (HYCEZBIO, HYCCK8) was added to each well. Following 1-hour incubation in the cell culture incubator, absorbance was measured at 450 nm using a microplate reader (BioTek, SynergyMx).

### 2.8 Colony formation assay

Ishikawa and KLE endometrial cancer cells were seeded in 6-well plates at a density of 1 × 10³ cells/well and cultured until individual colonies contained >50 cells. The colonies were then fixed with 4% paraformaldehyde for 20 minutes, stained with 1% crystal violet for 20 minutes, washed three times with PBS, and imaged.

### 2.9 Matrigel invasion test

A 24-well Transwell chamber (Corning Costar Transwell, Cambridge, MA, USA) with 8 μm pores was utilized for cell invasion assays. The chamber was pre-coated with 80 µl of Matrigel (diluted 1:8, BD Biosciences) and incubated at 37°C to allow gel formation through polymerization. The cells were transfected for 48 hours and then digested with trypsin. Next, cells were counted with a cell counting plate and the cell suspension was diluted to 2.5 × 10^5^ cells/ml with serum-deprived medium. 200 µl of cell suspension was placed in each upper chamber, while the lower chambers were filled with complete medium supplemented with 10% FBS, and incubated for 24 hours. Using a 400X inverted microscope, the count of invaded cells was quantified in three randomly chosen fields.

### 2.10 Scratch test

Endometrial cancer cells in logarithmic growth phase were digested with trypsin and then spread using six-well plates. After 24 hours of incubation, transfection was performed. After 48 h, a scratch was created with a 200 µl pipette tip, the samples were rinsed three times with PBS, serum-free 1640 or DMEM/F12 medium was added, and photographs were taken at 0 h, 24 h, 48 h, and 72 h.

### 2.11 Lentiviral transduction experiment

The shRNA and negative control lentiviruses used in this study were obtained from GeneChem (Shanghai,China).The target sequences for Ishikawa and KLE cells were 5’-GACCAUUCUAGUCUAAAUATT-3’ and 5’-AGGUUAUUGCAGGCUUUACUTT-3’,respectively, with a scrambled control sequence (5’-TTCTCCGAACGTGTCACGT-3’). Viral transduction was performed according to the manufacturer’s protocol. Briefly, KLE and Ishikawa cell lines were transduced with lentiviral particles. Following 10-hour incubation, the viral supernatant was replaced with complete medium. Transduction efficiency was evaluated 72 hours post-transduction by fluorescence microscopy. Stable cell clones were subsequently selected using 4 μg/mL puromycin.

### 2.12 Rescue experiments

SKL2001 was dissolved in DMSO. For rescue experiments, DDIAS-knockdown KLE and Ishikawa cells were treated with SKL2001 solution (MCE, Cat#HY-101085) at a final concentration of 20 μM for 24 hours, followed by Western blot analysis and subsequent functional assays.

### 2.13 Western Blot analysis

The determination of protein expression levels was achieved through the utilisation of the WB technique. The method was as follows: 1 × 10^6^ cells were inoculated in Petri dishes and incubated overnight. RIPA lysis buffer (Doctoral Biotechnology Co., Ltd., China) was used to extract total proteins in the cells after treatment. The proteins extracted from the cells were separated via SDS-PAGE, transferred to 0.22μm or 0.45μm PVDF membranes (Millipore) by membrane transfer, and incubated overnight at 4°C with the following antibodies: DDIAS (1:1500, Cat#M051478, Abmart, Shanghai, China), CyclinD1 (1:5000, Cat#60186–1-Ig, Proteintech, China), N-Cadherin (1:1000, Cat#CY5015, Abways, China), E-Cadherin (1:2000, Cat#CY1155, Abways, China), β-catenin (1:1000, Cat#CY3523, Abways, China), c-Myc (1:1000, Cat#CY5150. Abways, China), vimentin (1:1000, Cat#CY5134, Abways, China), survivin (1:1000, Cat#71G4B7, Cell Signaling Technology, Boston, USA), and β-actin (1:10000, Cat#AB0035, Abways China). After washing with TBST, the membranes were treated with anti-mouse or anti-rabbit secondary antibodies (Abways, China) and incubated at 37°C for 2 hours. The proteins were visualized via Pierce ECL substrate (Doctoral Biotechnology Co., Ltd., China) and detected via a bioimaging system (Bio-Rad, California, USA). The results were quantified as grey values via Image J software.

### 2.14 Statistical analysis

Statistical analysis was conducted using GraphPad Prism 9.5 software. Data are presented as mean ± standard deviations (x ± s). The Student’s t-test was applied to compare the differences between two groups. For comparisons between groups, ANOVA and Dunnett’s multiple comparisons were used. Each of the above experimental groups was repeated at least three times. A probability value of less than 0.05 is an demonstration of a statistically significant difference.

## 3. Results

### 3.1 DDIAS expression is elevated in EC and associated with poor patient prognosis

**Fig 1:**
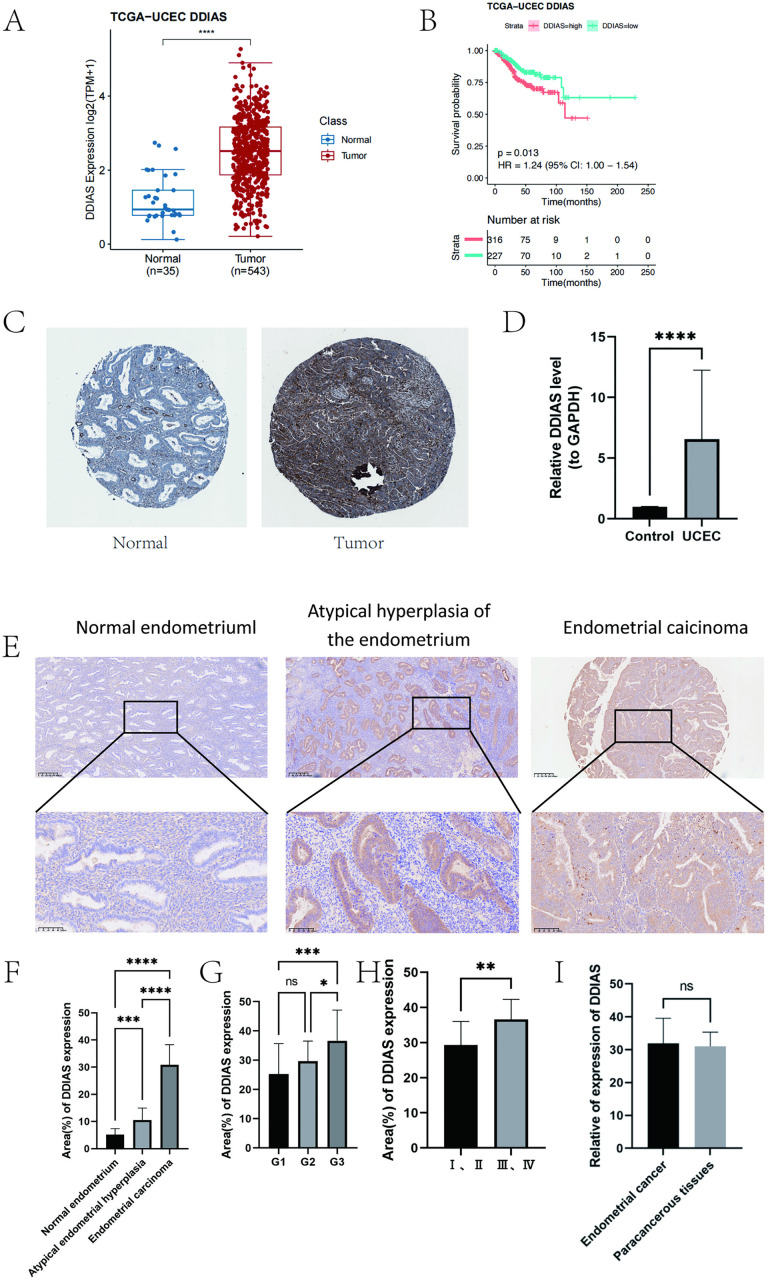
Expression of DDIAS in endometrial carcinoma tissues. **(A)** Transcriptional expression differences in DDIAS between endometrial cancer patients and normal controls. **(B)** Survival analysis plots for the low and high DDIAS expression groups. **(C)** Tissue expression of DDIAS in the endometrium from the HPA database. (Normal group on the left, tumor group on the right). **(D)** Expression levels of DDIAS mRNA in tumor tissues and normal tissues using the qPCR. **(E)**The expression levels of DDIAS in endometrial carcinoma tissues, atypical endometrial hyperplasia tissues and normal endometrium tissues using the immunohistochemistry. (F-I) Correlations between DDIAS and clinicopathological features. (compared with the control group, *: P < 0.05, **: P < 0.01, ***: P < 0.001, ****: P < 0.0001).

The results demonstrated that DDIAS mRNA expression levels were significantly greater in ECpatients compared to normal endometrial patients in the TCGA database (*P* < 0.0001) (**Fig 1A**). According to the expression level of DDIAS gene, it was categorized into two groups, the high expression group and the low expression group. A total of 227 EC patients were classified into the low-expression group, and 316 EC patients were classified into the high-expression group. The results revealed that the low expressing group survived significantly longer than the high expressing group(*P* = 0.013) (**Fig 1B**). Protein expression data for *DDIAS* obtained from the HPA database, also confirmed higher expression in tumor tissues (**Fig 1C**). In clinical samples, the expression levels of DDIAS were significantly elevated in tumor tissues compared to their normal counterparts(*P* < 0.0001) (**Fig 1D**).The staining results revealed that the DDIAS protein expression was positively expressed in the cytoplasm, with minimal expression in the nucleus. Immunohistochemical staining revealed that DDIAS was differentially expressed in normal endometrium tissues, atypical endometrial hyperplasia tissues and endometrial cancer tissues (**Fig 1E**). The level of DDIAS expression in endometrial cancer was markedly higher compared to both atypical endometrial hyperplasia and normal endometrial tissue(*P* < 0.0001), and the expression of DDIAS in atypical endometrial hyperplasia was significantly greater than that observed in normal endometrial tissue(*P* = 0.0043) (**Fig 1F**). Additionally, the statistical results revealed that in 116 endometrial cancers, the level of DDIAS positive expression showed a significant association with both histological grade and FIGO stage (*P* < 0.0001, *P* = 0.0013), independent of patient age and tumour size (*P* = 0.0815, *P* > 0.05). DDIAS expression was upregulated as the degree of differentiation decreased in endometrial cancer (**Fig 1G**). The expression of DDIAS was markedly elevated in patients diagnosed with stage III and IV endometrial carcinoma relative to those with stage I and II disease (**Fig 1H**). However, no significant difference in DDIAS protein expression levels was observed between tumor tissues and the corresponding paracancerous tissues(*P* = 0.5168) (**Fig 1I**).

### 3.2 DDIAS is highly expressed in KLE and Ishikawa cell

**Fig 2:**
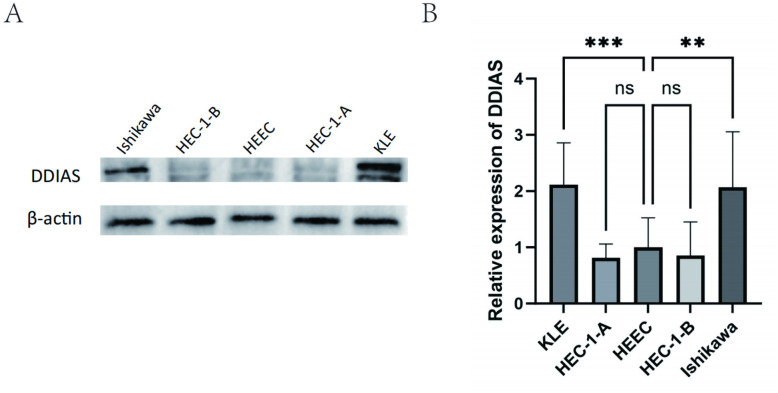
The protein expression levels of DDIAS in KLE, Ishikawa, HEEC, HEC-1-A and HEC-1-B cell. (compared with the control group, **: P < 0.01, ****: P < 0.0001). **(A-B)** The expression levels of DDIAS using the WB.

An examination was conducted of the expression of DDIAS in four endometrial cancer cell and in normal endometrial cell lines (HEECs) via WB (**Fig 2A**). The findings of the study demonstrated that the expression level of DDIAS protein was significantly higher in KLE and Ishikawa cells compared to HEECs (*P* = 0.0010, *P* = 0.0017). The expression of DDIAS was reduced in HEC-1-A and HEC-1-B cells relative to HEECs, although this difference was not statistically significant(*P* = 0.9066*, P* = 0.9575) (**Fig 2B**). For subsequent experiments, the KLE and Ishikawa cell lines were selected.

**Fig 3:**
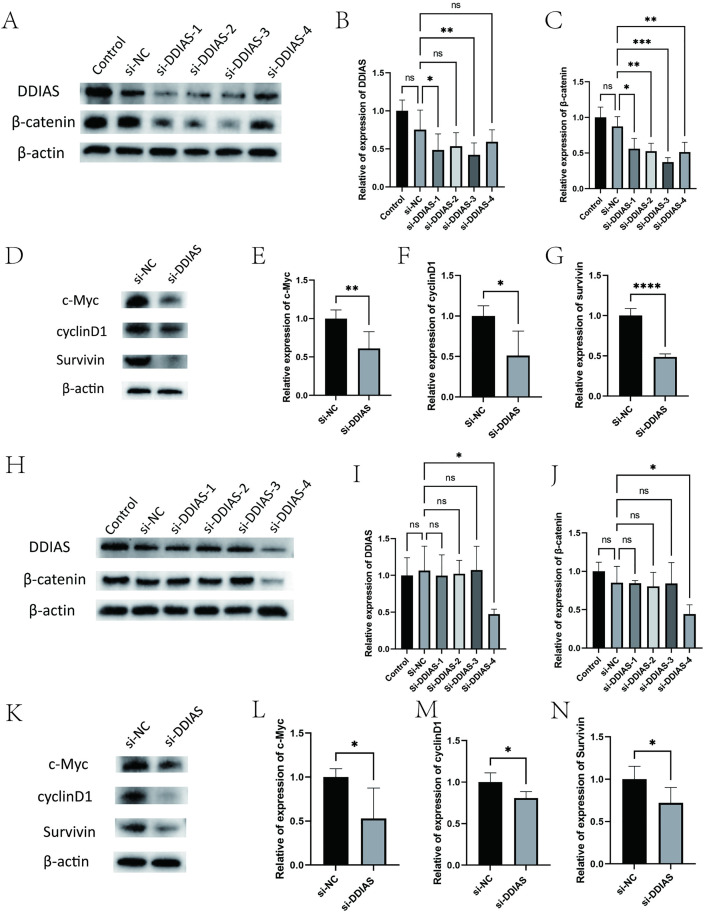
Expression levels of β-catenin pathway proteins in KLE and Ishikawa cells after knockdown of DDIAS (A-G) The expression levels of DDIAS, β-catenin, c-Myc, cyclin D1, survivinin in KLE cells with DDIAS knocked down. **(H-N)** The expression levels of DDIAS, β-catenin, c-Myc, cyclin D1, survivinin in Ishikawa cells with DDIAS knocked down. (compared with the control group, *: P < 0.05, **: P < 0.01, ***: P < 0.001, ****: P < 0.0001).

### 3.3 DDIAS knockdown in endometrial cancer cell lines inhibits the β-catenin pathway

The effect of knocking down DDIAS in KLE and Ishikawa cells was examined via WB. The results of the study revealed that, in KLE cells, DDIAS knockdown was greatest in the si-DDIAS-3 group (*P* = 0.0097) (Fig 3A-3B). Additionally, the β-catenin protein level in the si-DDIAS-3 group was significantly reduced compared to that in the si-NC group (*P* = 0.0002) (Fig 3C). The expression levels of the β-catenin pathway proteins, including c-Myc, cyclin D1, and survivin was reduced (*P* = 0.0070, *P* = 0.0104, *P* < 0.0001) (Fig 3D-3G). In Ishikawa cells, si-DDIAS-4 achieved the most effective DDIAS knockdown (*P* = 0.0139) (Fig 3H-3I), and the β-catenin protein level was found to be significantly reduced in the si-DDIAS-4 group compared to the si-NC group(*P* = 0.0002) (Fig 3J). The expression levels of the downstream proteins associated with the β-catenin pathway, namely c-Myc, cyclin D1 and survivin, was reduced(*P* = 0.0189, *P* = 0.0302, *P* = 0.0260) (Fig 3K-3N).

**Fig 4:**
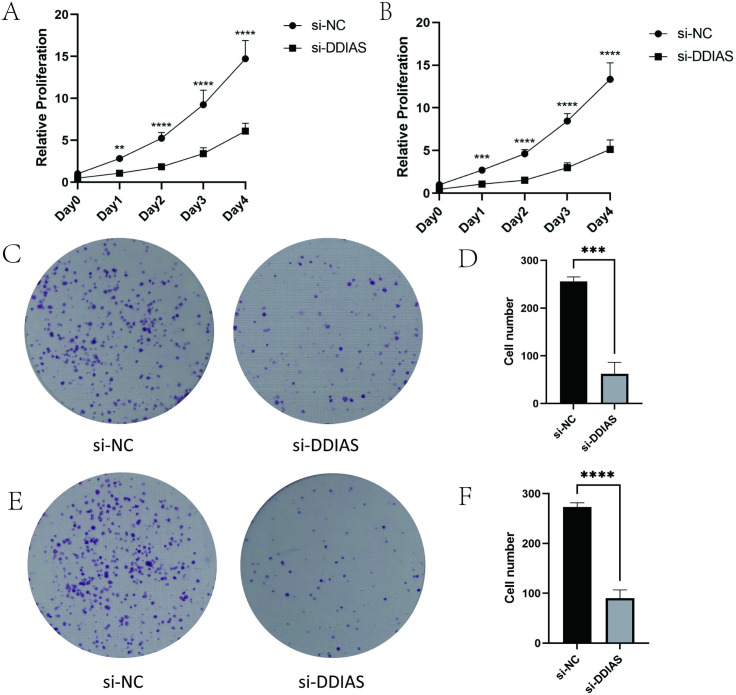
The proliferation capacities of KLE and Ishikawa cells with DDIAS knocked down. **(A)** Assessment of KLE cell viability using the CCK-8 assay. **(B)** Assessment of Ishikawa cell viability using the CCK-8 assay. **(C-D)** Assessment of KLE cell viability using the colony formation assay. **(E-F)** Assessment of Ishikawa cell viability using the colony formation assay.(compared with the control group, **: P < 0.01, ***: P < 0.001, ****: P < 0.0001).

### 3.4 DDIAS knockdown attenuates the proliferation of KLE and Ishikawa cells

CCK-8 assay results demonstrated that DDIAS knockdown significantly attenuated the proliferative capacity of both KLE and Ishikawa cells (**Fig 4A**–**4B**). Colony formation assays consistently revealed that genetic silencing of DDIAS markedly reduced the clonogenic potential of KLE and Ishikawa cell lines (KLE, *P* = 0.0002 and Ishikawa, *P* < 0.0001) (**Fig 4C**–**4F**).

### 3.5 DDIAS knockdown attenuates the migration and invasion of KLE and Ishikawa cells

**Fig 5:**
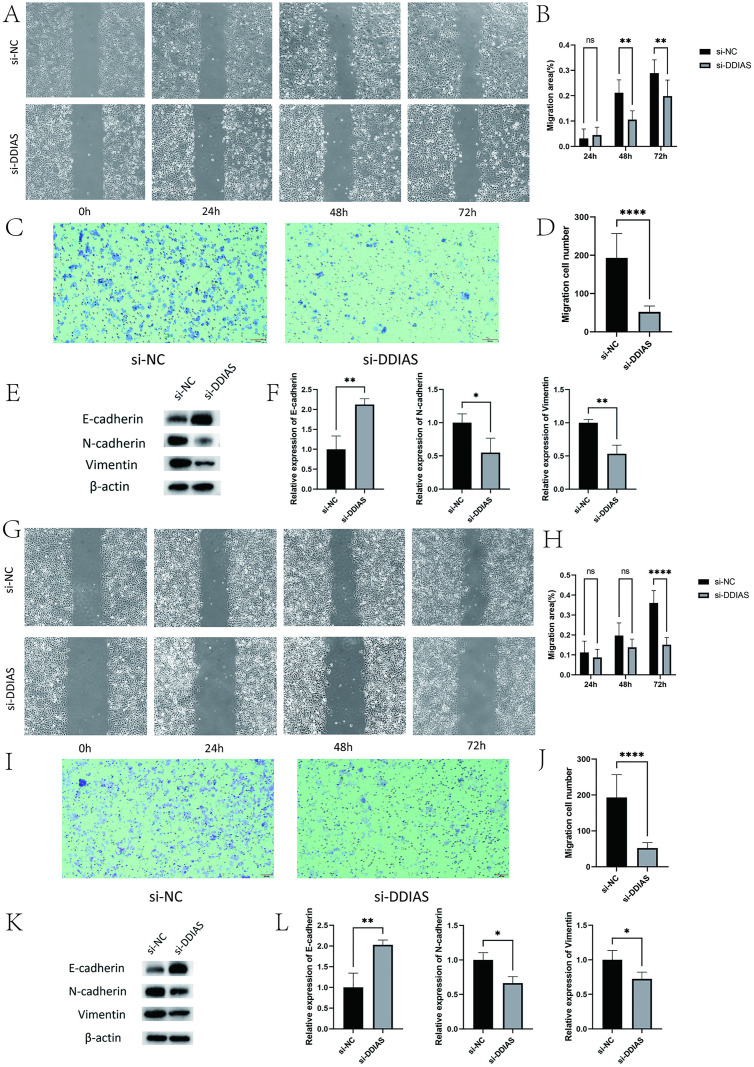
The invasion and migration capacities of KLE and Ishikawa cells with DDIAS knocked down. **(A-D)** The migration and invasion capacities of KLE cells with DDIAS knocked down. **(E-F)** The expression levels of EMT-related proteins in KLE cells with DDIAS knocked down.**(G-J)** The migration and invasion capacities of Ishikawa cells with DDIAS knocked down. **(K-L)** The expression levels of EMT-related proteins in Ishikawa cells with DDIAS knocked down. (compared with the control group, *: P < 0.05, **: P < 0.01, ****: P < 0.0001).

The results of the scratch and Transwell assays revealed that the migratory and invasive properties of KLE and Ishikawa cells were decreased after DDIAS knockdown (KLE, *P* = 0.0057, *P* < 0.0001 and Ishikawa, *P* < 0.0001, *P* < 0.0001) (Fig 5A–5D) (Fig 5G–5J). The WB results revealed that the EMT process in KLE and Ishikawa cells was inhibited after DDIAS knockdown, with E-cadherin levels exhibiting a significant increase, N-cadherin and vimentin levels exhibiting a significant decrease compared to those observed in the si-NC group (KLE, *P* = 0.0059, *P* = 0.0484, *P* = 0.0043 and Ishikawa, *P* = 0.0081, *P* = 0.0148, *P* = 0.0456)(Fig 5E–5F)(Fig 5K–5L).

### 3.6 The β-catenin agonist SKL2001 rescues EMT inhibition caused by DDIAS knockdown

**Fig 6:**
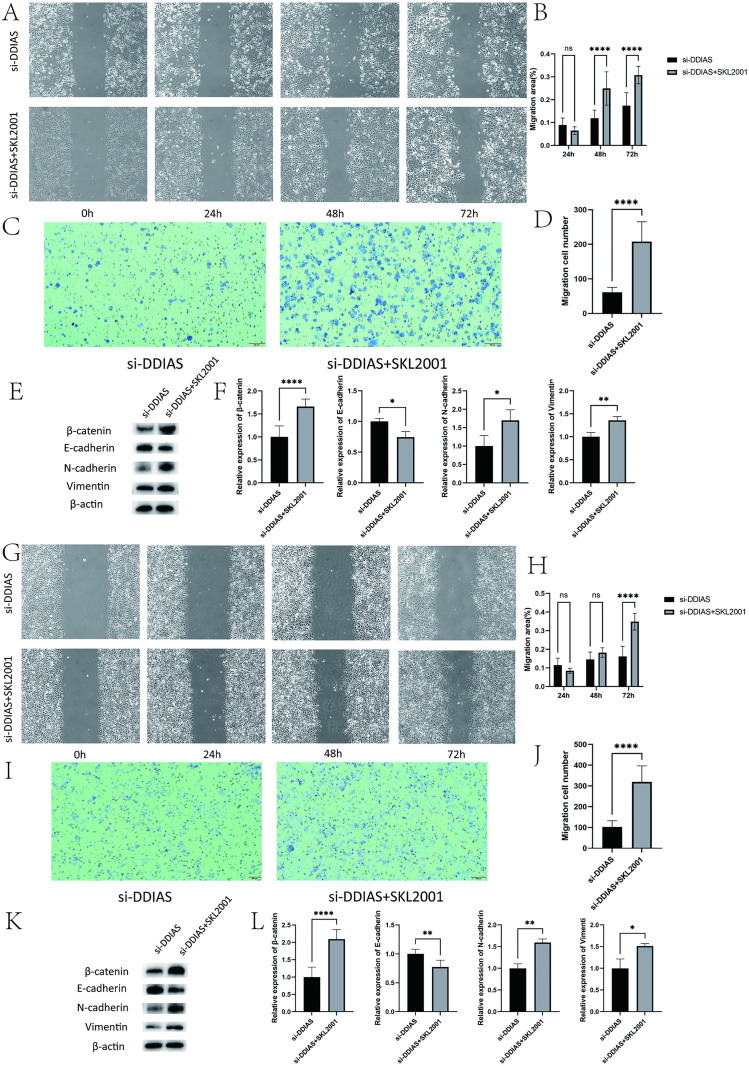
Effect of β-catenin agonist SKL2001 on the migration and invasion of KLE and Ishikawa cells with DDIAS knockdown. (A-D) The migration and invasion capacities of KLE cells. (E-F) The expression levels of EMT-related proteins in KLE cells. (G-J)The migration and invasion capacities of Ishikawa cells. (K-L) The expression levels of EMT-related proteins in Ishikawa cells. (compared with the control group, *: P < 0.05, **: P < 0.01, ****: P < 0.0001).

Endometrial cancer cells with DDIAS knocked down were treated with the β-catenin agonist SKL2001, and the results of the scratch and Transwell assays suggested that the migration and invasive ability of both types of endometrial cancer cells were enhanced (KLE, *P* < 0.0001, *P* < 0.0001 and Ishikawa, *P* < 0.0001, *P* < 0.0001) (Fig 6A–6D)(Fig 6G–6J). The findings of the WB assay indicated that the suppression of EMT was restored, with E-cadherin levels in the SKL2001 group being considerably lower than those observed in the si-DDIAS group(KLE, *P* = 0.0018 and Ishikawa, *P* = 0.0031), while N-cadherin and vimentin levels were found to be significantly elevated in the SKL2001 group relative to the si-DDIAS group (KLE, *P* = 0.0410, *P* = 0.0205 and Ishikawa, *P* = 0.0015, *P* = 0.0162) (Fig 6E–6F) (Fig 6K–6L).

### 3.7 Successful DDIAS knockdown in lentivirus-transduced cells

**Fig 7:**
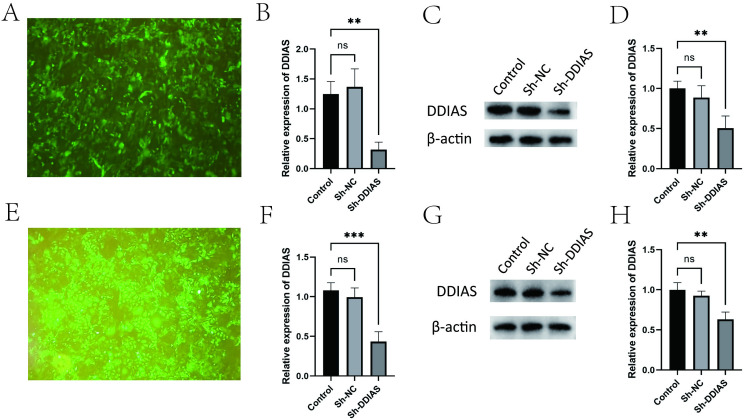
Lentiviral transduction experiment in KLE cell and Ishikawa cell. **(A)** Fluorescence microscopy images of KLE cell post viral transfection. **(B)**Expression levels of DDIAS mRNA in KLE cell using the qPCR. **(C-D)** The expression levels of DDIAS in KLE cell using the WB. **(E)** Fluorescence microscopy images of Ishikawa cell post viral transfection. **(F)** Expression levels of DDIAS mRNA in Ishikawa cell using the qPCR. **(G-H)** The expression levels of DDIAS in Ishikawa cell using the WB. (compared with the control group, **: P < 0.01, ***: P < 0.001).

Following lentiviral transduction and puromycin selection, fluorescence microscopy confirmed efficient transduction in KLE cells (**Fig 7A**). Quantitative analysis demonstrated significant downregulation of DDIAS in transduced cells compared to both blank control and negative control groups. Specifically: qPCR revealed a reduction in DDIAS mRNA expression (vs blank control: P = 0.0054; vs scramble control: P = 0.0029) (**Fig 7B**). WB analysis showed a decrease in DDIAS protein levels (vs blank control: P = 0.0094; vs scramble control: P = 0.0298) (**Fig 7C**–**7D**). Fluorescence microscopy confirmed successful lentiviral transduction in Ishikawa cells (**Fig 7E**). Comparative analyses demonstrated significant DDIAS suppression at both transcriptional and translational levels: qPCR revealed a reduction in DDIAS mRNA expression (vs blank control: P = 0.0011; vs scramble control: P = 0.0022). Western blot quantification showed decrease in protein abundance (vs blank: P = 0.0007; vs scramble: P = 0.0038) (**Figs 7F**–**7H**).

## 4. Discussion

Since the recurrence, metastasis, and highly invasive nature of advanced EC pose a serious threat to patients, exploring new therapeutic targets is of significant clinical importance. Identifying new genes involved in regulating endometrial cancer progression is an important approach for identifying therapeutic targets. DDIAS is expressed at a high level in various cancers, including hepatocellular carcinoma, lung cancer, cervical cancer, breast cancer, and glioma, and is positively associated with poor prognosis [5–8]. This study concluded from public database analysis that DDIAS is highly expressed in endometrial cancer compared to normal tissue and is strongly associated with poor prognosis. The present study found DDIAS to be highly expressed in endometrial cancer and atypical endometrial hyperplasia, and the difference in expression between these two types of DDIAS was statistically significant. In endometrial cancer, higher expression levels of DDIAS have been positively correlated with increased tumor grade and advanced FIGO stage. Moreover, the analysis of the database indicated that patients exhibiting elevated DDIAS expression demonstrated a suboptimal prognosis. In our study, DDIAS was identified for the first time as an oncogene for endometrial cancer.

Recent studies have demonstrated that DDIAS can drive tumor progression and metastasis via multiple mechanisms, including DNA synthesis and repair, p53 signalling, proliferation and metastasis, STAT3 activation, death ligand signalling, and drug resistance [8]. Consistent with the oncogenic role of DDIAS, DDIAS knockdown using siRNA markedly suppressed the migration and invasion of endometrial cancer cells in our studies.

The Wnt/β-catenin signaling pathway controls multiple cellular processes, including proliferation, differentiation, apoptosis, in addition to a variety of biological behaviours in cancer cells, including cell migration and invasion [12,13]. There is a robust correlation between hyperactivation of the Wnt/β-catenin pathway and endometrial cancer [14–16]. It is evident that β-catenin plays a pivotal role in the canonical Wnt signalling pathway, serving as a crucial mediator in the process of intercellular adhesion junctions [17]. During normal physiology, β-catenin is localized at the cell membrane and binds to E-cadherin and α-catenin at the junctions between epithelial cells to maintain epithelial cell integrity [18]. Abnormal activation of the intracellular Wnt/β-catenin pathway results in the nuclear translocation of β-catenin, which subsequently interacts with transcription factors of the T-cell-specific factor (TCF)/lymphoid enhancer binding factor (LEF) family. This binding activates specific Cyclin D1, C-myc, MMP-7 and other target genes, which have been implicated in tumourigenesis and metastasis [13]. In HeLa cells, the ERK5/MEF2B signaling pathway is activated upon epidermal growth factor (EGF) stimulation, leading to upregulation of DDIAS expression. This process further promotes cancer cell invasion via the β-catenin pathway. Notably, knockdown of DDIAS suppresses the transcriptional activity of β-catenin/TCF, indicating a critical role of DDIAS in mediating β-catenin-dependent oncogenic signaling [19]. In lung cancer, DDIAS exerts a cytoprotective effect against DNA-damaging agents and TNF-related apoptosis-inducing ligand (TRAIL)-mediated cytotoxicity. Furthermore, it positively regulates cancer cell invasion by stabilizing β-catenin [8]. In gliomas, LEF1 has been identified as a downstream factor of DDIAS, and DDIAS knockdown modulates LEF1 to inhibit glioma cell proliferation, invasion, migration and tumour growth [8]. LEF1, a member of the TCF/LEF transcription factor family, is critically involved in the β-catenin signaling pathway [20]. Consistent with our results, the results demonstrate a reduction in the protein expression of β-catenin and the related pathway proteins c-Myc, cyclin D1 and survivin following DDIAS knockdown.

The process of Epithelial-mesenchymal transition (EMT) is characterised by the loss of polarity in epithelial cells and the subsequent acquisition of motility and invasive characteristics typical of mesenchymal cells, making it one of the risk factors affecting tumour prognosis [21]. E-cadherin is a crucial molecule for epithelial intercellular adhesion, and the absence of its expression in the course of EMT serves as a key indicator of EMT initiation. Conversely, the upregulation of mesenchymal markers, such as N-cadherin and vimentin, reflects the acquisition of mesenchymal characteristics [22].

EMT involves a variety of signalling pathways, including the nuclear factor-κB-related pathway, the Notch, Ras, and Wnt/β-catenin pathways [23–26]. Extensive research has highlighted the pivotal role of epithelial-mesenchymal transition (EMT) in the development and progression of endometrial cancer, including its involvement in tumourigenesis, metastasis, resistance to apoptosis, and invasion [27]. However, the mechanisms that regulate EMT have not been well studied. Among them, it is hypothesised that the Wnt/β-catenin protein exerts a regulatory influence on the process of EMT[28]. In HeLa cells, knockdown of DDIAS inhibited the expression of proteins MMP1, MMP3, and COX2, which are EMT markers [19]. In gliomas, DDIAS downregulation significantly suppressed mRNA of vimentin expression [8]. In our experiments, knockdown of DDIAS suppressed EMT in endometrial cancer cells, whereas EMT inhibition was rescued upon administration of the β-catenin agonist SKL2001. Therefore, we hypothesized that DDIAS mediates EMT and promotes endometrial carcinogenesis and development through the β-catenin signalling pathway.

## 5. Conclusions

To summarise, the present study revealed that DDIAS mediates EMT in endometrial cancer cells via the β-catenin pathway, thus promoting endometrial cancer cell migration and invasion. DDIAS may be a potential target for targeted therapies in endometrial cancer patients, but the molecular mechanism involving DDIAS and β-catenin must be further investigated.

## Supporting information

S1 File The raw images of western blot. (ZIP)
