# Supplementary figures and images for: DDIAS promotes endometrial cancer progression via β-catenin signaling
Source: PLoS One. 2025 Oct 6;20(10):e0331851. doi: 10.1371/journal.pone.0331851 (PMC12500153; doi:10.1371/journal.pone.0331851)

# uncropped gels for Figure 2A

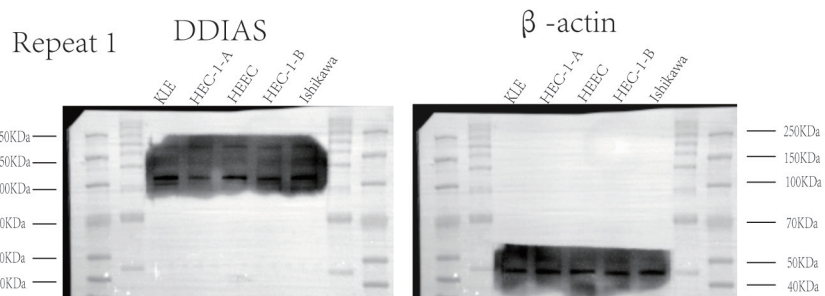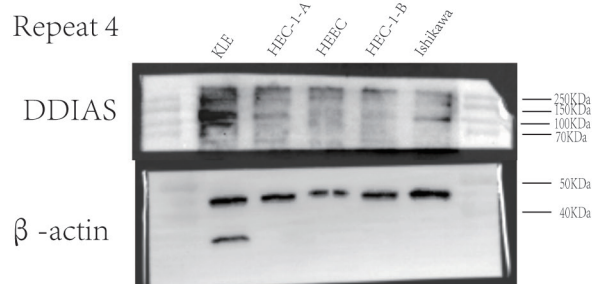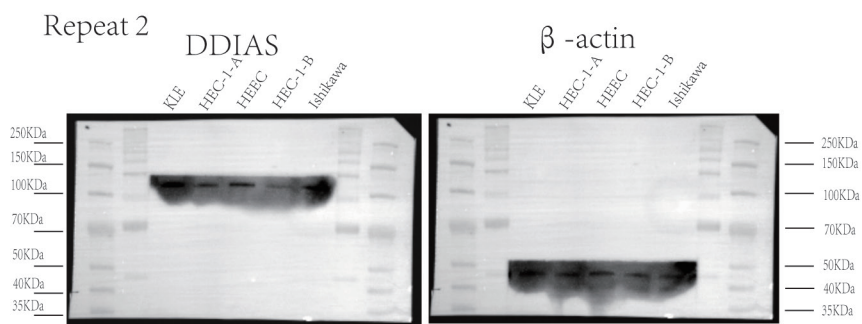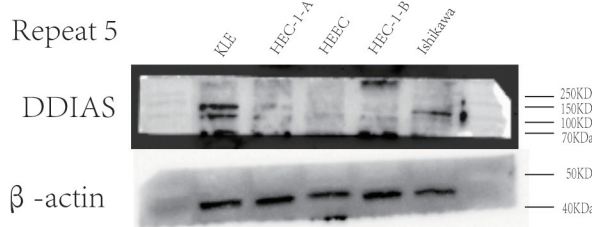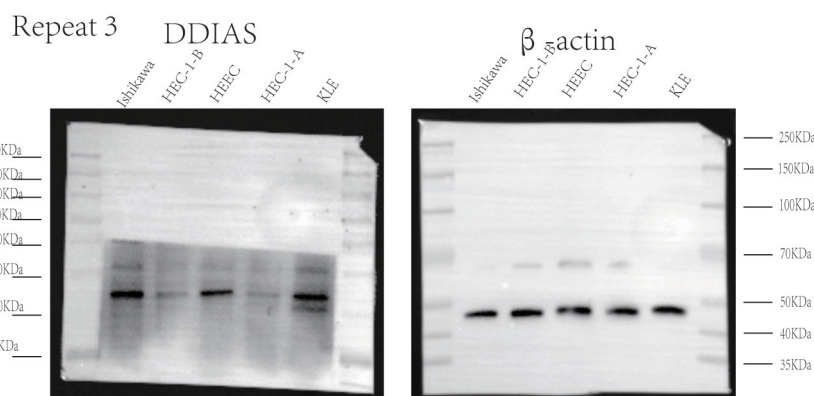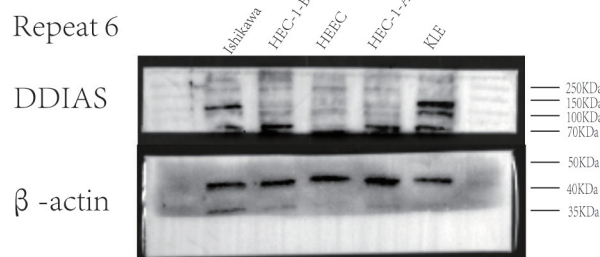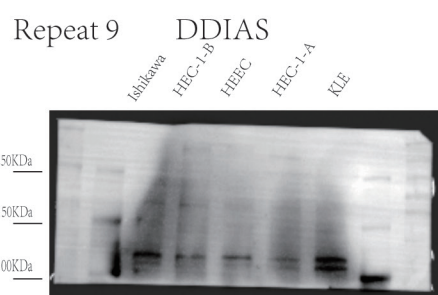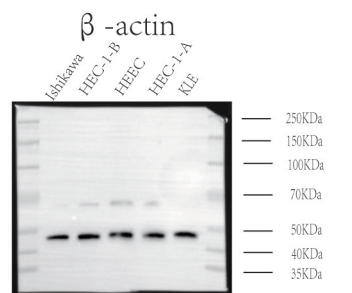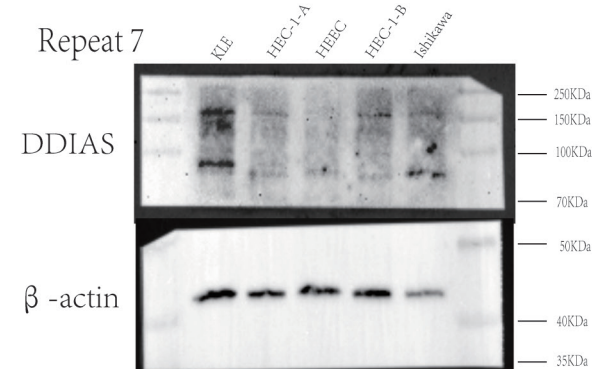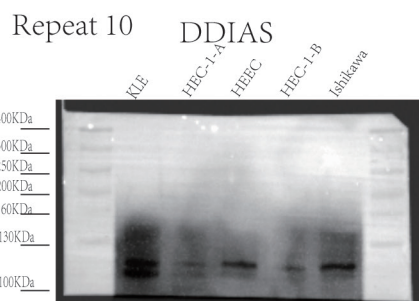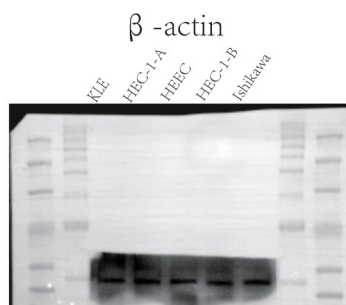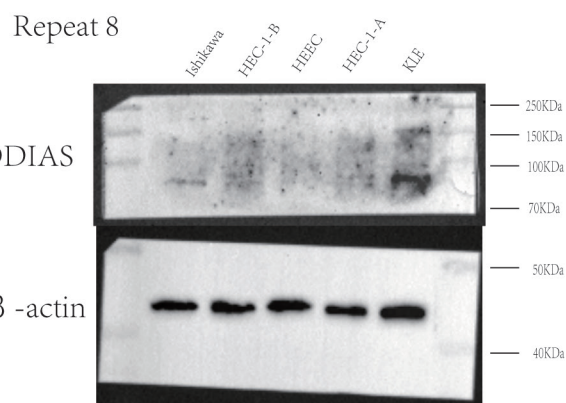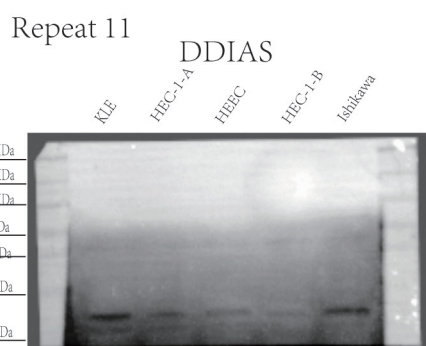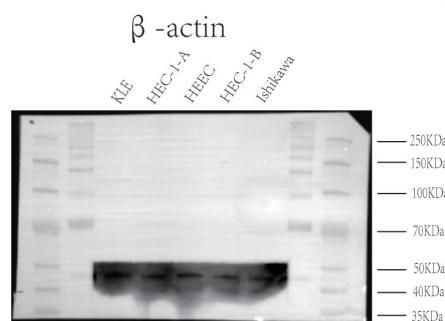

Supplement: S1 File — (ZIP) [file pone.0331851.s001.zip › S1 File. The raw images of western blot/Figure 2A.pdf]

## DDIAS

DDIAS

$\beta$ -actin

$\beta$ -catenin

$\beta$ -catenin

$\beta$ -actin

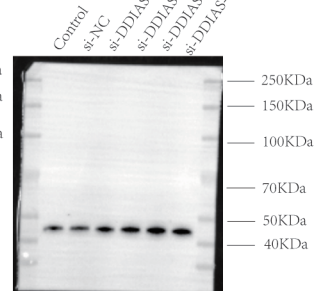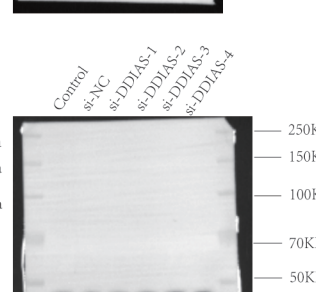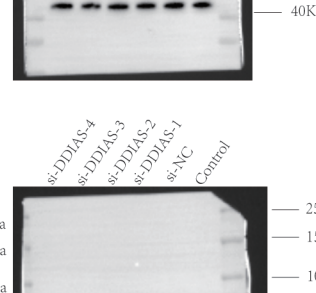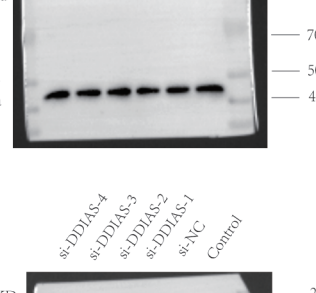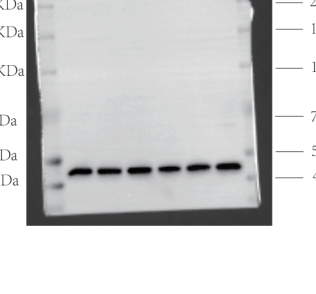

Supplement: S1 File — (ZIP) [file pone.0331851.s001.zip › S1 File. The raw images of western blot/Figure 3A.pdf]

uncropped gels for Figure3H  
DDIAS

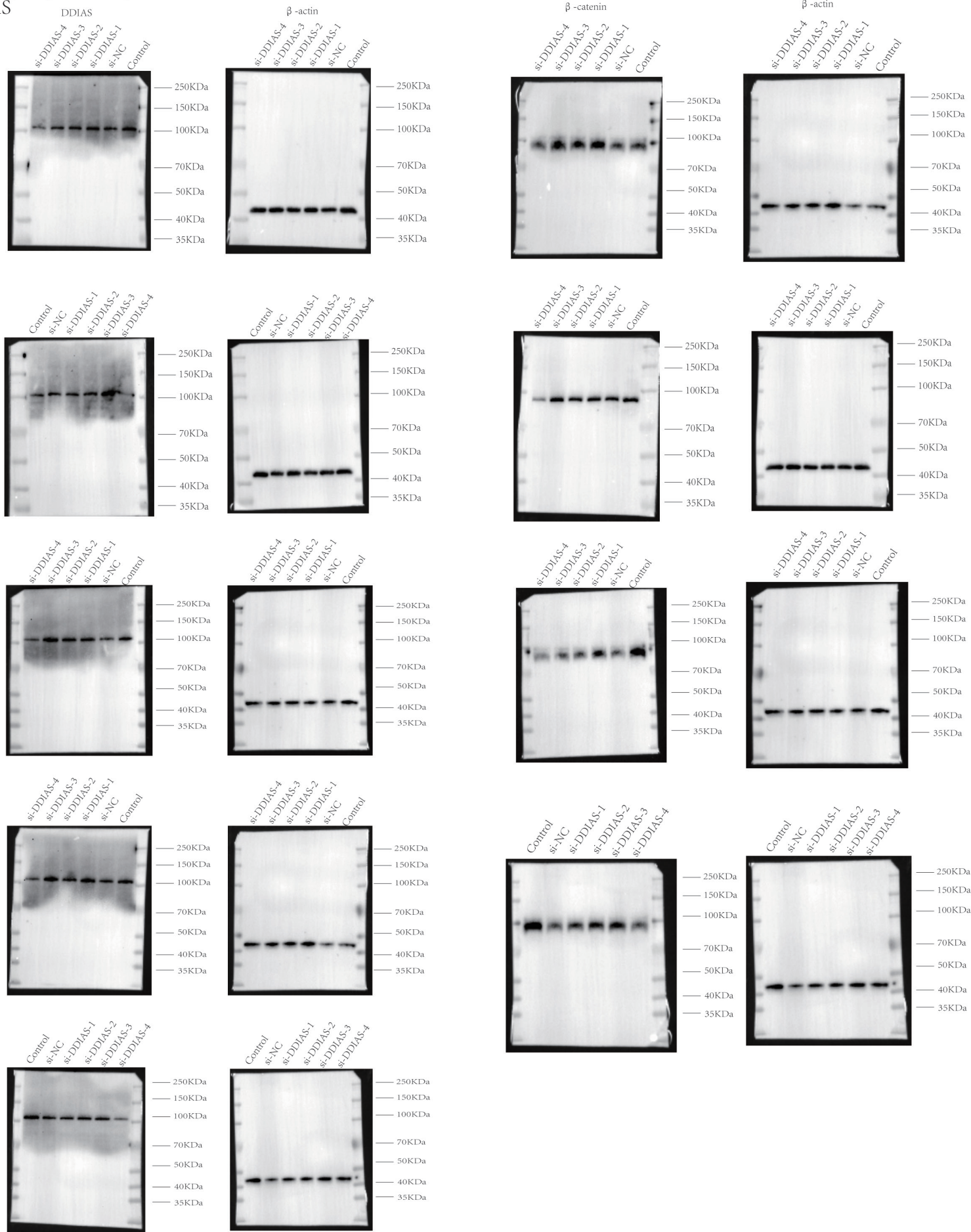

Supplement: S1 File — (ZIP) [file pone.0331851.s001.zip › S1 File. The raw images of western blot/Figure 3H.pdf]

c-Myc

cyclinD1

survivin

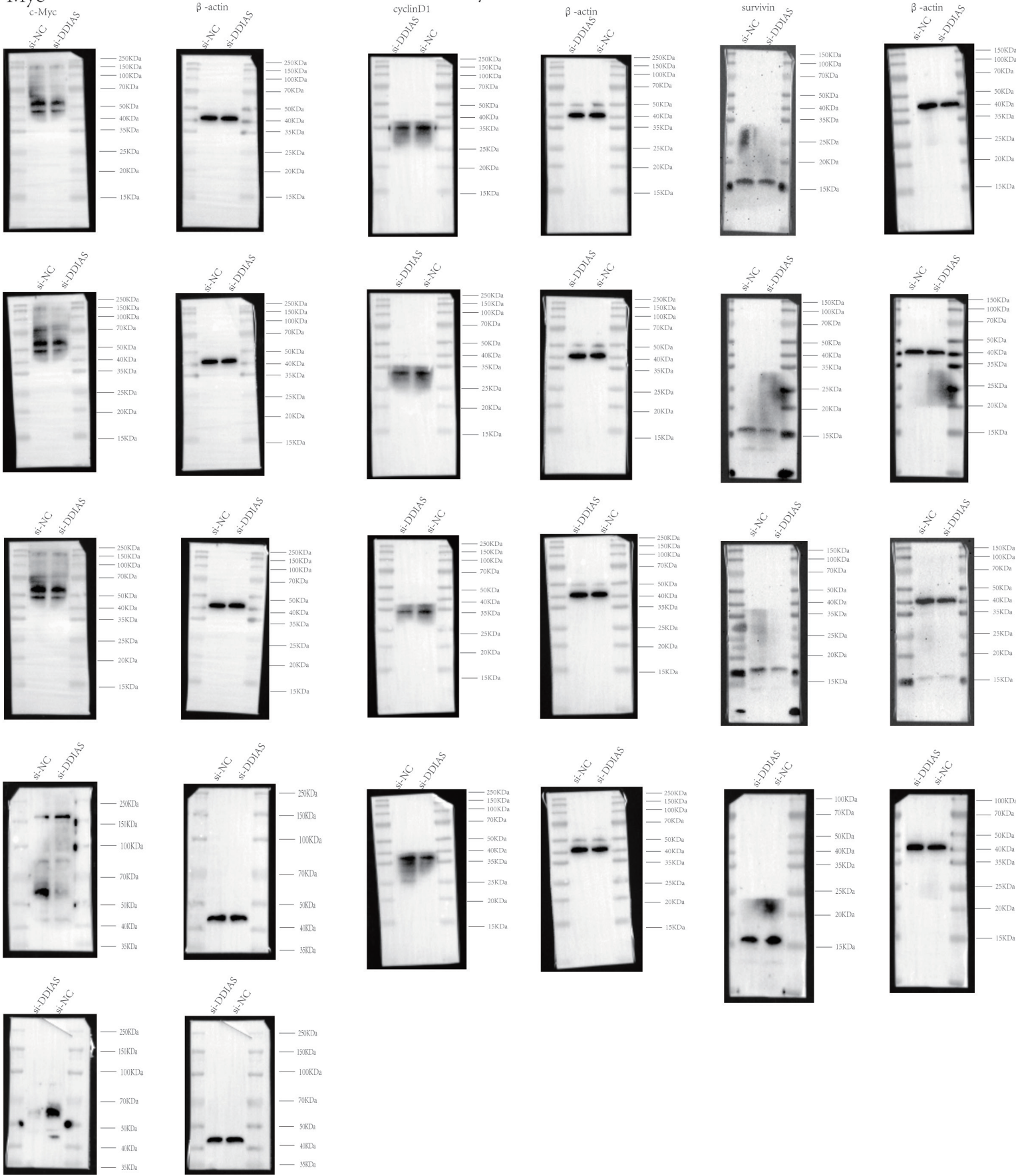

Supplement: S1 File — (ZIP) [file pone.0331851.s001.zip › S1 File. The raw images of western blot/Figure 3K.pdf]

uncropped gels for Figure5E

E-cadherin

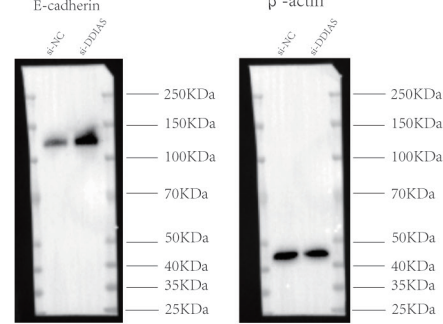

N-cadherin

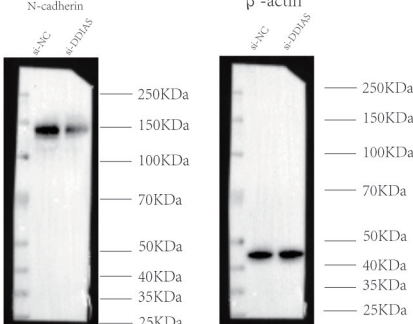

vimentin

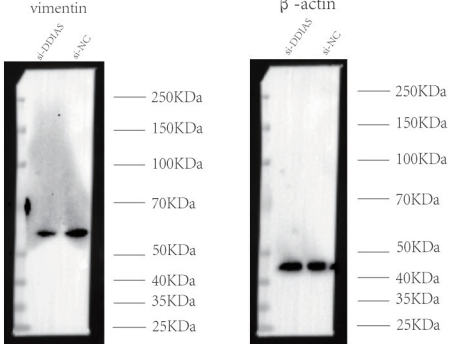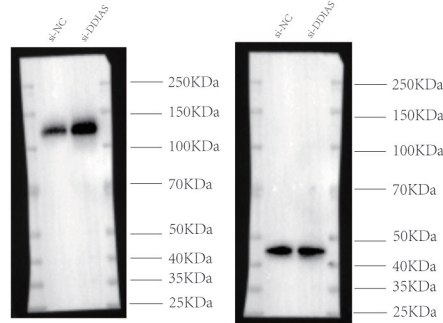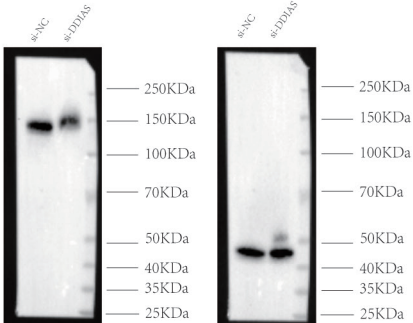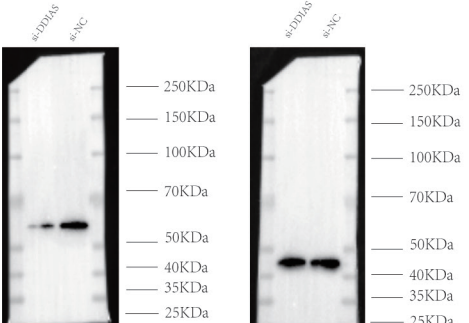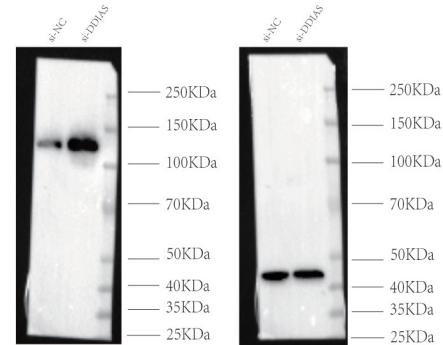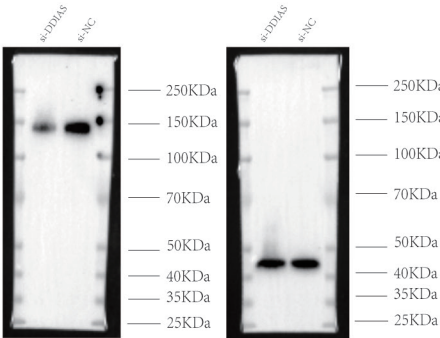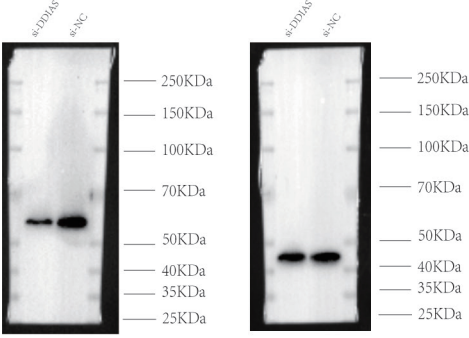

Supplement: S1 File — (ZIP) [file pone.0331851.s001.zip › S1 File. The raw images of western blot/Figure 5E.pdf]

uncropped gels for Figure5K

## E-cadherin

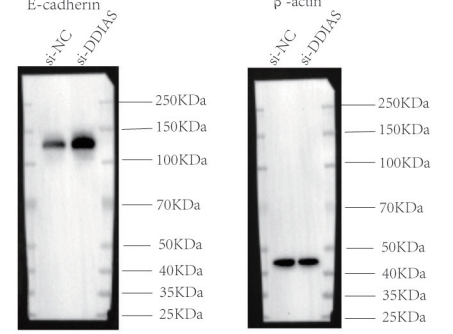

## N-cadherin

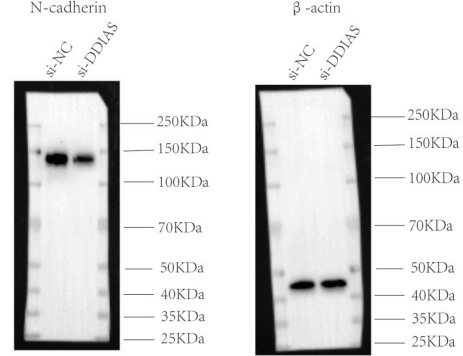

## vimentin

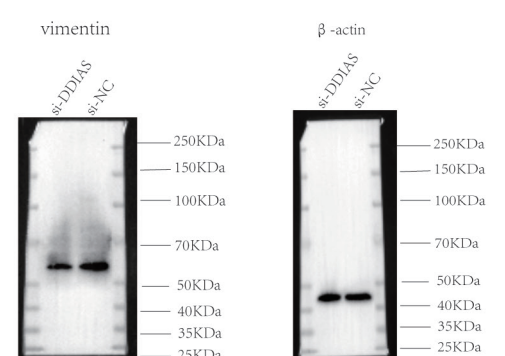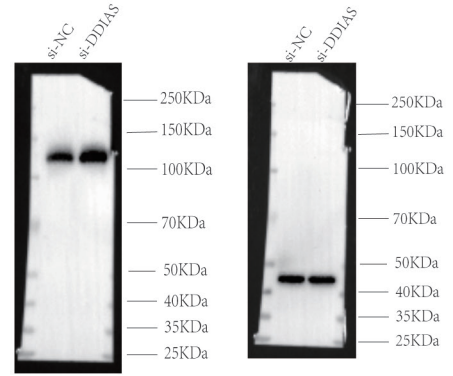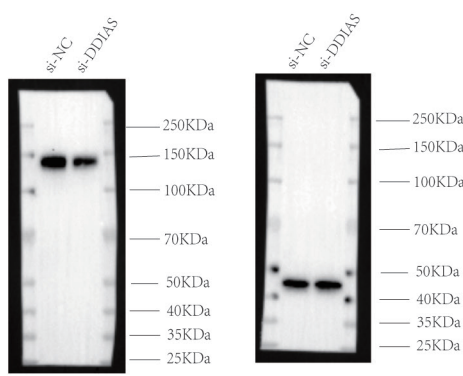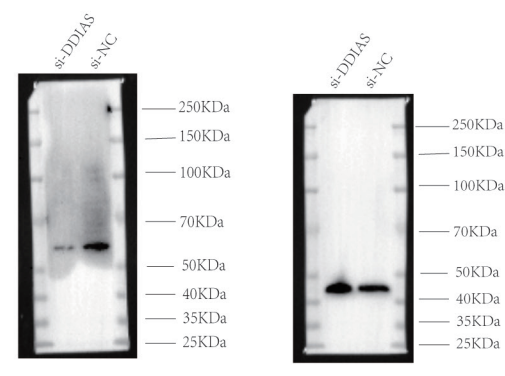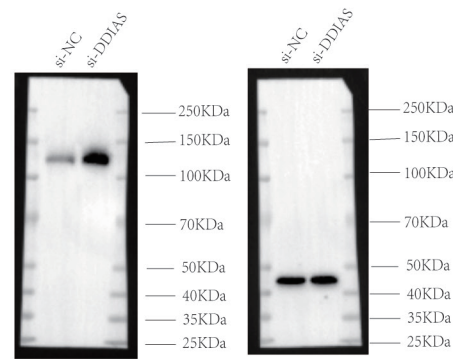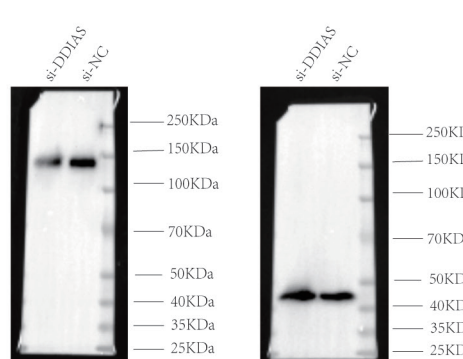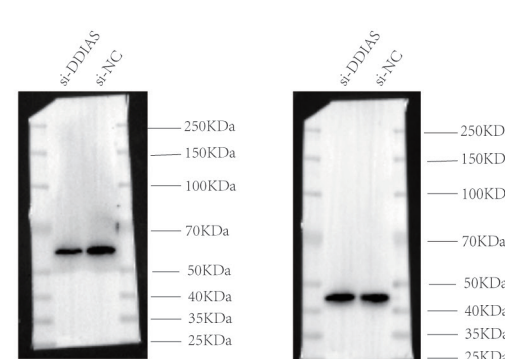

Supplement: S1 File — (ZIP) [file pone.0331851.s001.zip › S1 File. The raw images of western blot/Figure 5K.pdf]

# uncropped gels for Figure6E

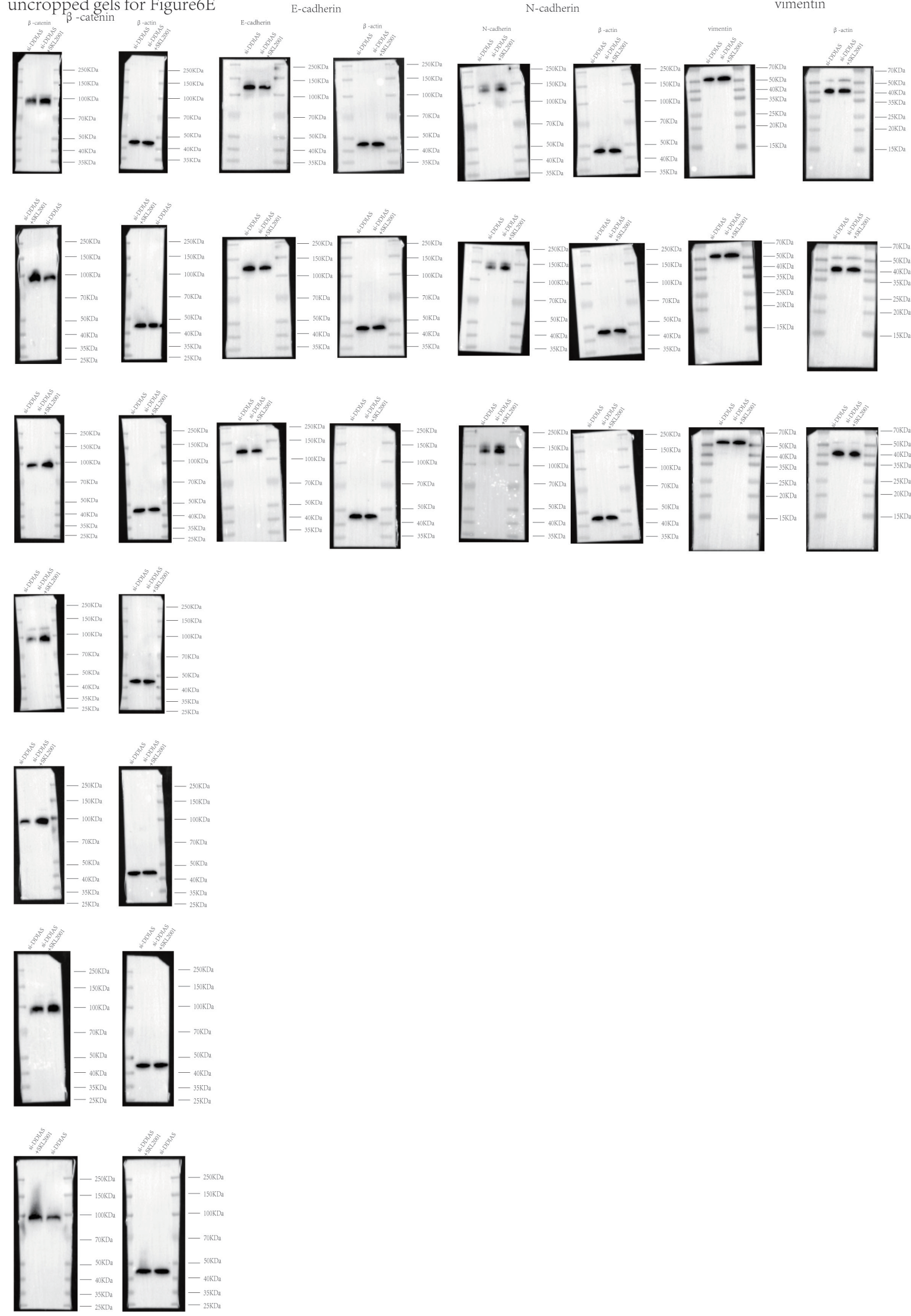

Supplement: S1 File — (ZIP) [file pone.0331851.s001.zip › S1 File. The raw images of western blot/Figure 6E.pdf]

# uncropped gels for Figure6K

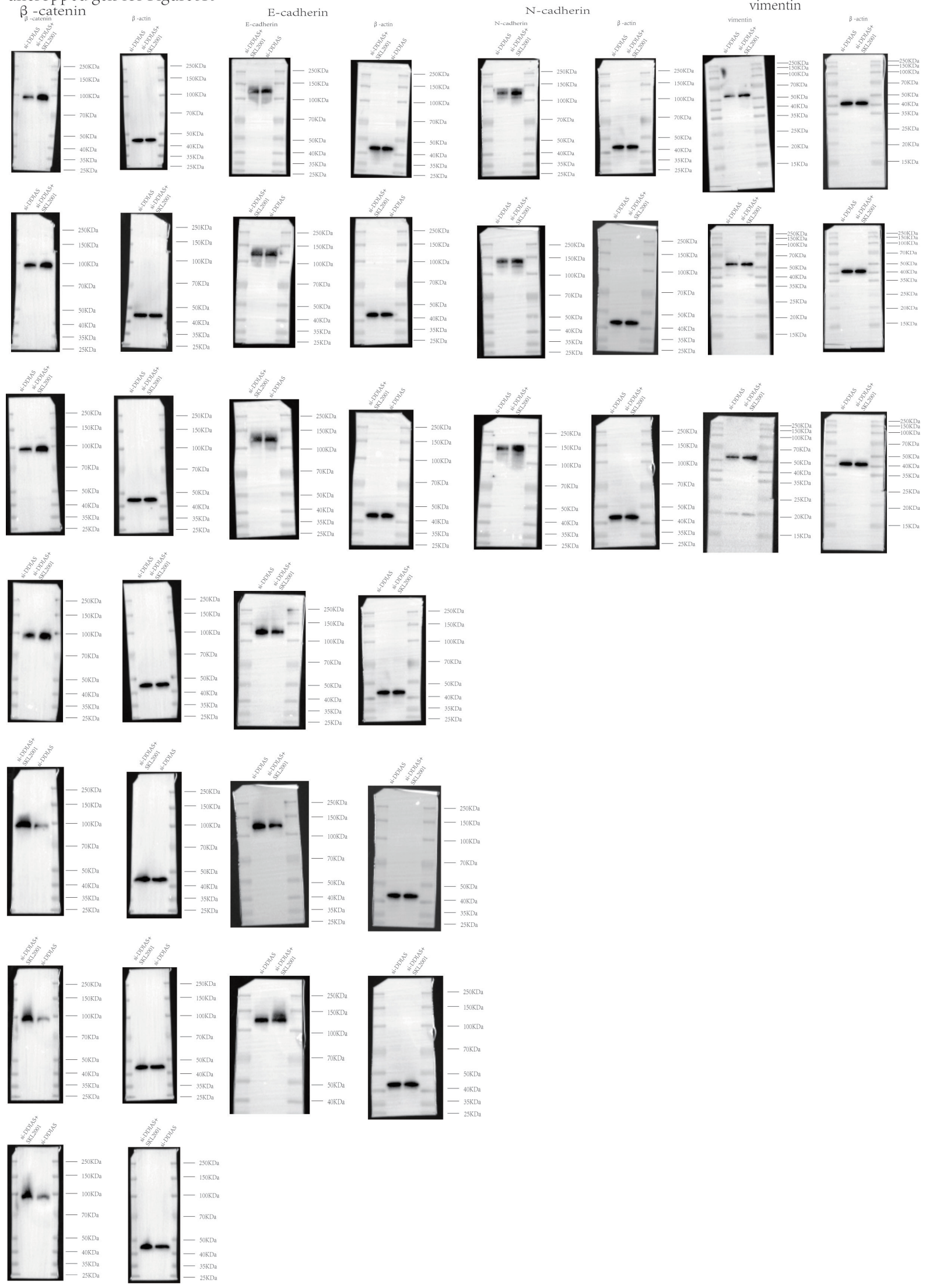

Supplement: S1 File — (ZIP) [file pone.0331851.s001.zip › S1 File. The raw images of western blot/Figure 6K.pdf]

uncropped gels for Figure7C

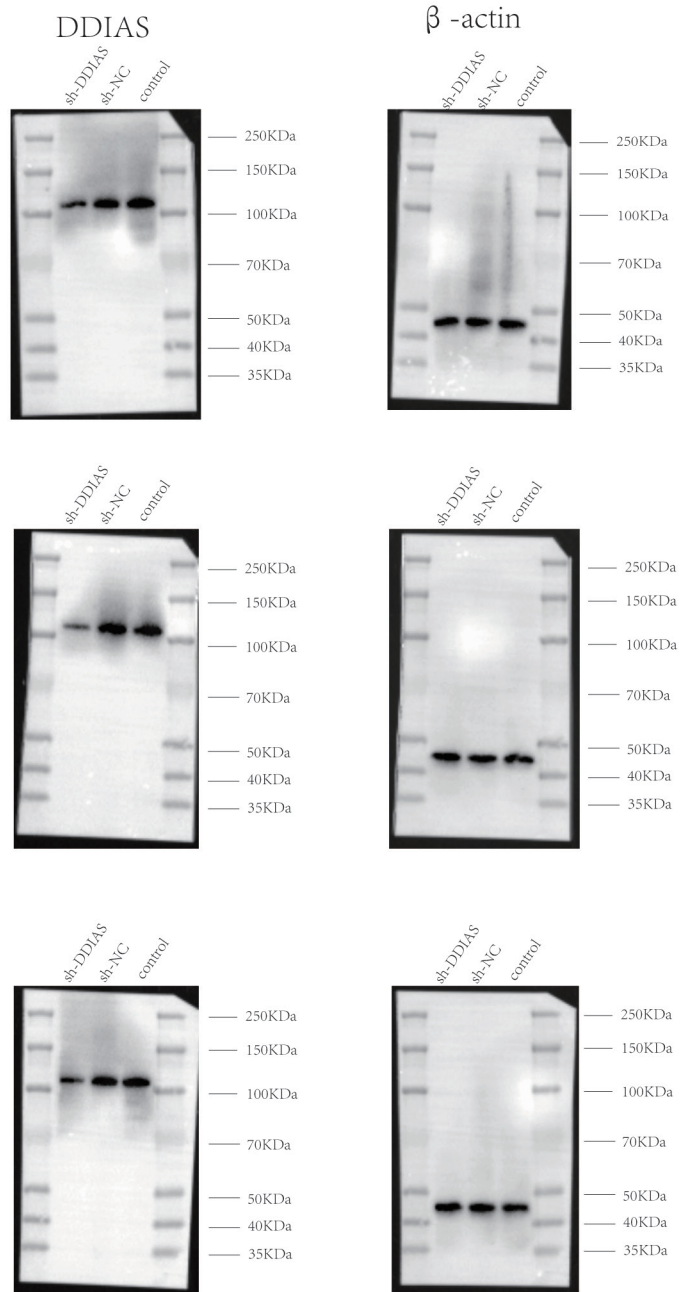

uncropped gels for Figure7G

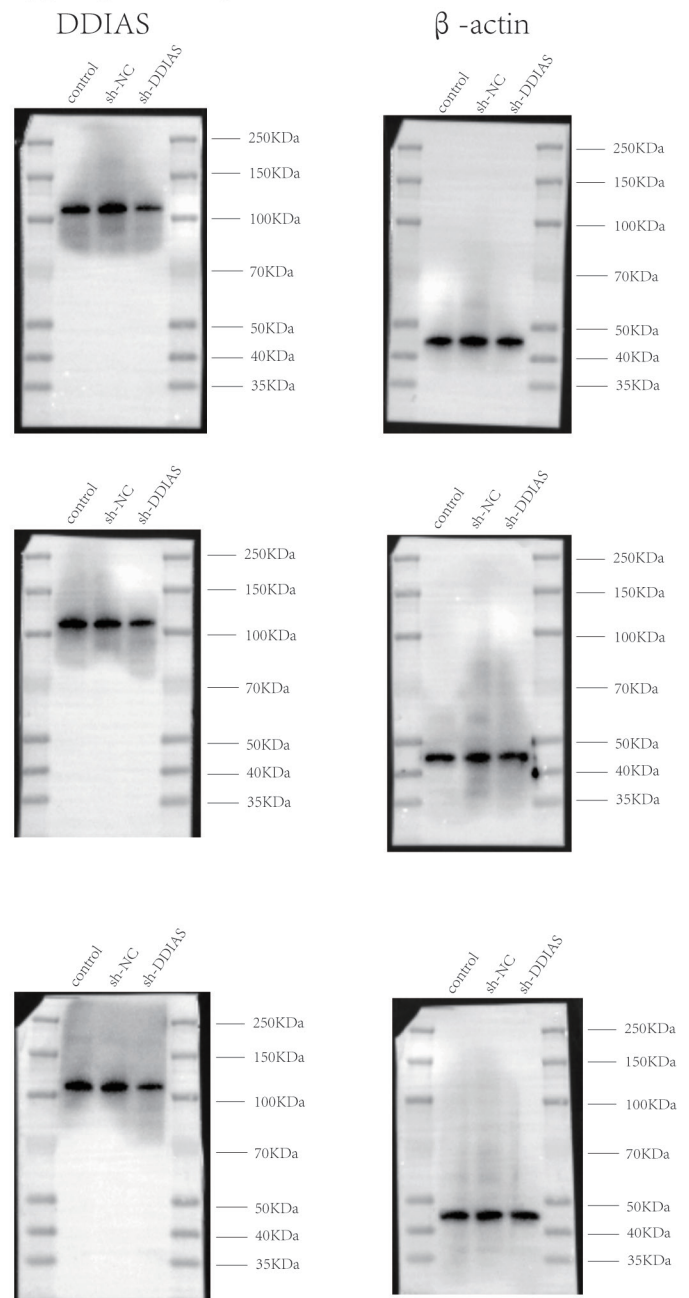

Supplement: S1 File — (ZIP) [file pone.0331851.s001.zip › S1 File. The raw images of western blot/Figure 7.pdf]
