# Supplementary material for: DDIAS promotes endometrial cancer progression via β-catenin signaling
Source: PLoS One. 2025 Oct 6;20(10):e0331851. doi: 10.1371/journal.pone.0331851 (PMC12500153; doi:10.1371/journal.pone.0331851)

uncropped gels for Figure3D

C-Myc

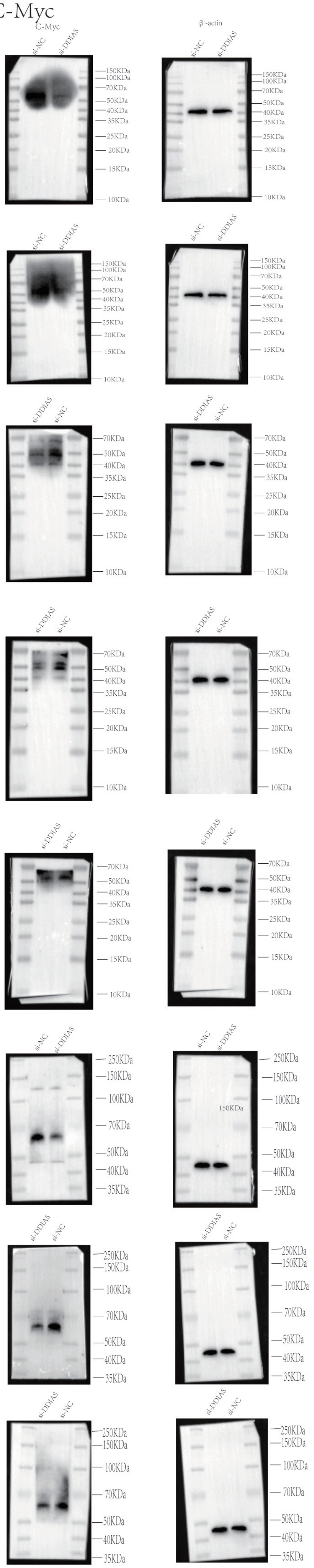

cyclinD1

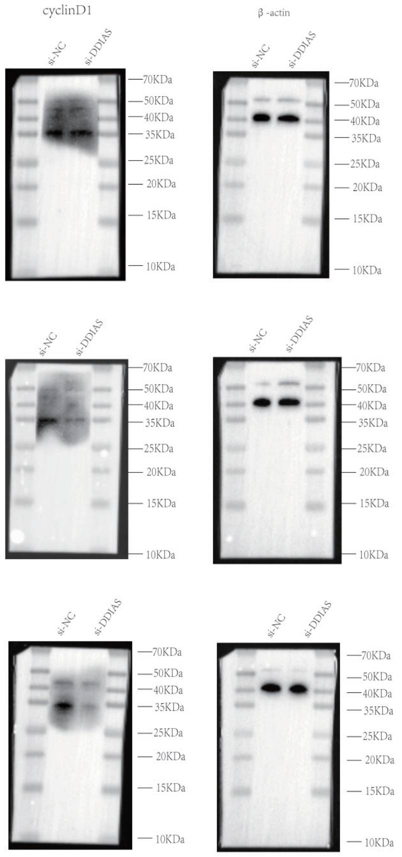

survivin

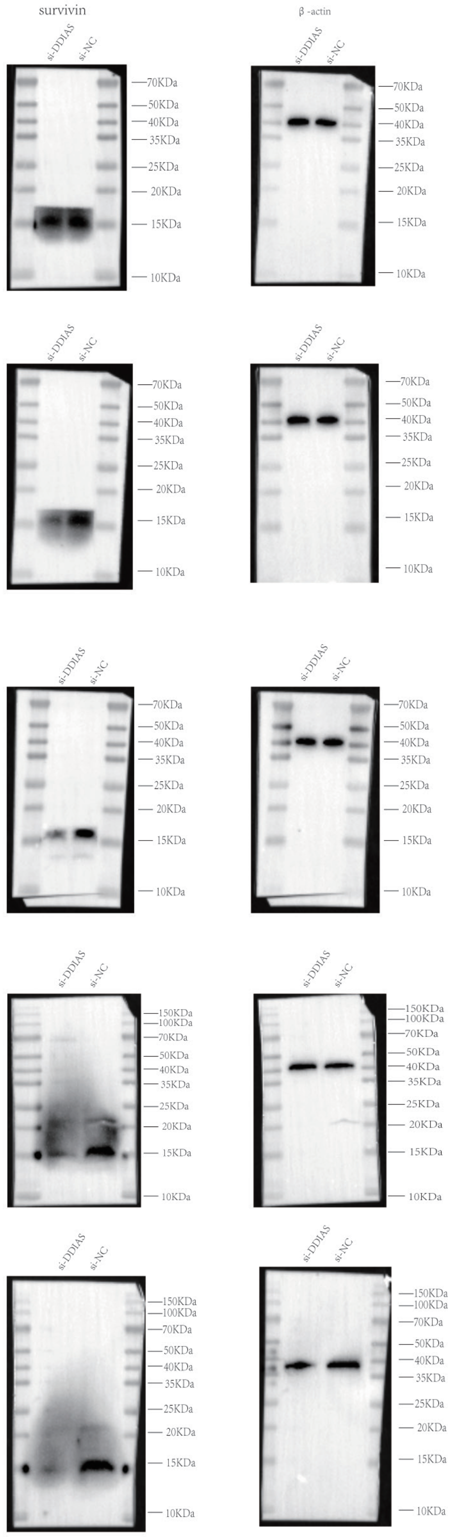

Supplement: S1 File — (ZIP) [file Figure 3D.pdf › S1 File. The raw images of western blot/Figure 3D.pdf]
